# Plasma KL-6 reflects pulmonary severity and longitudinal response to enzyme replacement therapy in acid sphingomyelinase deficiency type B

**DOI:** 10.1016/j.ymgmr.2026.101343

**Published:** 2026-07-30

**Authors:** Karla Cifuentes-Uribe, Nathalie Guffon, Lucie Boulière, Ségolène Turquier, Cécile Acquaviva-Bourdain, Roseline Froissart, Magali Pettazzoni

**Affiliations:** a National Reference Centre for Hereditary Metabolic Diseases, Hospices Civils de Lyon, France; b Department of Biochemistry and Molecular Biology, Lyon University Hospital, France; c Department of Pulmonary Function Testing, Louis Pradel Hospital, Hospices Civils de Lyon, France

**Keywords:** Acid sphingomyelinase deficiency, Alveolar epithelial injury, Biomarker, Enzyme replacement therapy, Interstitial lung disease, KL-6, Pulmonary diffusing capacity

## Abstract

Acid sphingomyelinase deficiency (ASMD) type B is frequently complicated by interstitial lung disease without validated circulating pulmonary biomarkers. In this retrospective longitudinal study of five patients treated with enzyme replacement therapy, plasma KL-6 was evaluated in relation to CT-based severity, DLCO, and treatment response. Higher pre-treatment KL-6 concentrations were observed with higher exploratory CT severity grades, and KL-6 levels generally decreased or remained stable during follow-up. A transient rise occurred during SARS-CoV-2 infection. KL-6 may reflect dynamic alveolar epithelial injury in ASMD.

## 1 Introduction

Acid sphingomyelinase deficiency (ASMD) is an autosomal recessive lysosomal storage disorder caused by pathogenic variants in the *SMPD1* gene, resulting in deficient acid sphingomyelinase activity and accumulation of sphingomyelin within lysosomes [1]. ASMD type B is characterized by hepatosplenomegaly, dyslipidemia, and progressive interstitial lung disease (ILD), which represents a major contributor to morbidity [2], [3], [4], [5], [6], [7]. Pulmonary monitoring relies on diffusing capacity for carbon monoxide (DLCO) and chest computed tomography (CT). However, DLCO may be difficult to perform in younger or cognitively impaired patients, and it is non-specific for ILD [8]. Plasma lysosphingomyelin (lysoSM) correlates with systemic disease severity [9], [10] and decreases in response to enzyme replacement therapy (ERT) [11], but it is not lung-specific and has not been validated to capture pulmonary disease activity. Although ERT with olipudase alfa improves pulmonary function in patients with ASMD, no circulating biomarker has been formally validated to reflect overall lung disease burden. Krebs von den Lungen-6 (KL-6) is a high-molecular-weight glycoprotein corresponding to the extracellular domain of mucin-1 (MUC1), expressed on type II alveolar pneumocytes. Elevated plasma KL-6 levels were reported in interstitial lung diseases and reflect alveolar epithelial injury and regeneration [12], [13]. Its relevance in ASMD has not previously been evaluated. We therefore investigated the relationship between KL-6, pulmonary severity, and longitudinal response to ERT in patients with ASMD type B.

## 2 Methods

This retrospective single-center study included 5 patients (1 child and 4 adults) with genetically and enzymatically confirmed ASMD type B, followed at our national reference center. All patients received ERT and underwent regular pulmonary and biochemical assessments.

Baseline was defined as the last available measurement obtained prior to ERT initiation (time < 0 years). One patient did not have pre-treatment KL-6 measurements and was excluded from baseline analyses. Plasma KL-6 was measured using a chemiluminescent immunoassay (Lumipulse® G KL-6, Fujirebio). A concentration of 500 U/mL, a commonly used clinical decision threshold in the KL-6 literature [14], was used for descriptive interpretation. Healthy controls were not included, and no study-specific reference interval was established.

Plasma lysosphingomyelin (LysoSM) was measured as previously described [15]. Pulmonary involvement was evaluated by DLCO (% predicted) and chest CT. A study-specific global CT severity grade (0–3) was assigned based on review of routine chest CT reports. Grade 0 was reserved for a normal CT but was not represented in this cohort, whereas grades 1–3 reflected increasing overall severity of the interstitial and parenchymal abnormalities described in the reports. The component-specific semiquantitative HRCT scoring system used in ASMD clinical trials [11] was not applied because the CT images had not undergone standardized rereading. This internal, non-validated grade was used only for exploratory correlation analyses. Given the small sample size, analyses were exploratory. Baseline associations between KL-6 and pulmonary severity were assessed using Spearman's rank correlation coefficient. Longitudinal intra-individual associations between KL-6 and DLCO were evaluated using subject-mean–centered correlations to account for repeated measures within individuals.

## 3 Results

### 3.1 Baseline correlation between KL-6 and pulmonary severity

**Fig. 1:**
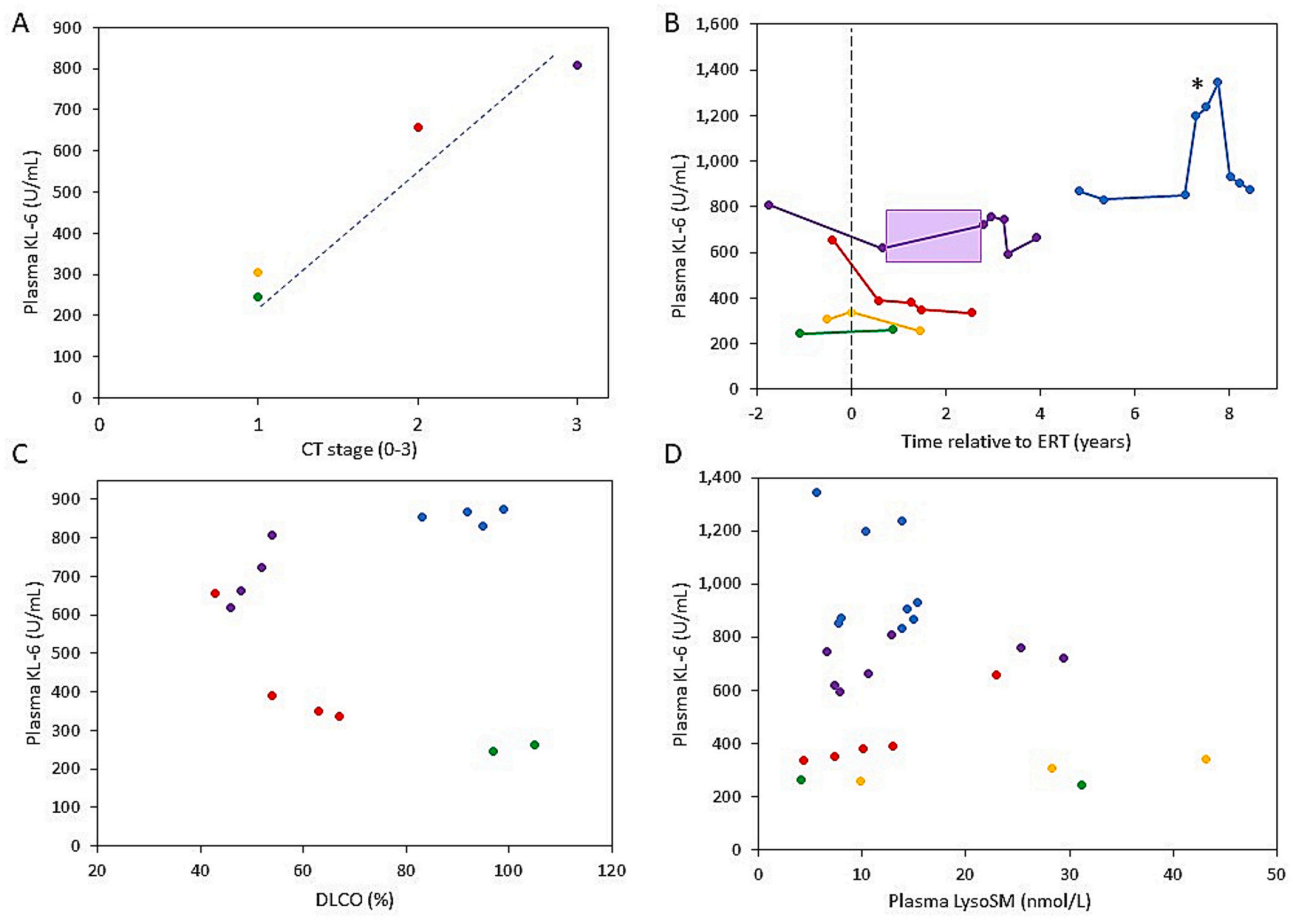
Plasma KL-6 before and during enzyme replacement therapy in ASMD type B. (A) Pre-treatment association between plasma KL-6 and the study-specific global CT severity grade. Baseline is defined as the last available measurement obtained prior to ERT initiation (time < 0 years). (B) Longitudinal evolution of plasma KL-6 relative to ERT initiation (time 0). The shaded area indicates a period of ERT interruption. The asterisk indicates a documented SARS-CoV-2 infection. (C) Relationship between plasma KL-6 and DLCO, % predicted, across repeated measurements. (D) Relationship between plasma KL-6 and plasma lysosphingomyelin (LysoSM, *N* < 1.2 nmol/L) across repeated measurements. Patients are color-coded consistently across all panels.

Four patients had available pre-treatment KL-6 measurements. Higher baseline KL-6 concentrations were observed in patients with more severe pulmonary involvement (Fig. 1A). The study-specific CT severity grades were 1 in two patients, 2 in one patient, and 3 in one patient; no patient had a normal CT (grade 0). Pre-treatment KL-6 levels showed a strong monotonic association with the exploratory CT severity grade (Spearman ρ = 0.95, *p* = 0.051), indicating that higher plasma KL-6 concentrations were associated with greater radiological severity in this small sample.

Among the three patients with available pre-treatment DLCO values, higher KL-6 levels were associated with lower DLCO% predicted (Spearman ρ = −0.50, *p* = 0.667). Although limited by sample size, these findings suggest that KL-6 reflects structural and functional pulmonary severity in ASMD type B.

### 3.2 Longitudinal evolution under enzyme replacement therapy

A total of 26 KL-6 measurements were available across five patients during follow-up (Fig. 1B). Overall, KL-6 levels decreased or were stable after ERT initiation, although inter-individual variability was observed. Across repeated measurements, KL-6 fluctuations tended to inversely parallel DLCO changes (Fig. 1C). Within-subject analysis demonstrated an inverse association between KL-6 and DLCO (*r* = −0.34, *p* = 0.23; 14 paired observations across four patients), indicating that increases in KL-6 were directionally consistent with declines in gas transfer capacity. Despite the absence of pre-treatment KL-6 data in one patient, longitudinal follow-up provided informative observations, including a transient increase in KL-6 during a documented SARS-CoV-2 infection, concomitant with a mild decline in DLCO; both parameters subsequently improved after recovery. In another patient, KL-6 levels increased during a two-year interruption of ERT, whereas DLCO remained stable, and further increased following a documented SARS-CoV-2 infection. KL-6 also varied dynamically over time in relation to LysoSM levels (*r* = 0.17, *p* = 0.42), although this association was less pronounced.

## 4 Discussion

This study provides preliminary evidence that plasma KL-6 is associated with pulmonary disease severity and longitudinal changes during ERT in ASMD type B.

The observed association between pre-treatment KL-6 and the study-specific CT severity grade suggests a relationship with structural lung involvement. The inverse relationship with DLCO was directionally consistent with functional impairment. However, because these analyses included only four and three patients, respectively, and did not reach statistical significance, they should be interpreted cautiously.

Mechanistically, sphingomyelin accumulation in ASMD is associated with altered macrophage function and crystalline lung inflammation [16]. Pulmonary involvement in ASMD can affect both alveolar and interstitial compartments [17]. This inflammatory environment may contribute to repeated injury and regeneration of type II pneumocytes. KL-6 is released by injured and regenerating type II pneumocytes [12], [13] and should therefore be interpreted as a marker of associated alveolar epithelial injury rather than as a direct marker of lysosomal sphingomyelin storage. CT severity was assessed retrospectively using a study-specific global grade derived from routine radiology reports, rather than the component-specific HRCT score used in ASMD clinical trials [11], which requires standardized image rereading. In particular, the extent of ground-glass opacities was not systematically quantified. The association between KL-6 and CT severity should therefore be considered exploratory.

Under ERT, reduction of sphingomyelin burden is expected to attenuate macrophage activation and inflammatory signaling [18]. The decline in KL-6 observed in most patients is consistent with, but does not establish, a reduction in epithelial stress. The inverse intra-individual association between KL-6 and DLCO may reflect dynamic changes in alveolar epithelial integrity rather than fixed structural damage alone.

Interestingly, KL-6 may provide lung-oriented information not captured by systemic lysoSM levels, although larger studies are needed to confirm this dissociation. The transient KL-6 increase observed during SARS-CoV-2 infection further supports this interpretation. Viral infections induce diffuse alveolar epithelial injury, particularly affecting type II pneumocytes [19], [20], [21]. Increased KL-6 during this episode, followed by normalization after recovery, supports the concept that KL-6 reflects active epithelial injury in both chronic lipid-driven interstitial involvement and acute inflammatory insults. In the era of personalized medicine, detailed stratification of patients according to pulmonary severity is of major interest. A recent study suggested that analysis of exhaled breath could help identify biomarkers of lung involvement in ASMD [22]. KL-6 is a quantitative, non-invasive marker from routine plasma samples, representing a practical advantage. This study has major limitations, including the very small sample size, retrospective design, absence of healthy controls, and use of a non-validated CT grade derived from routine reports. Only four patients had pre-treatment KL-6 measurements. These results should therefore be considered hypothesis-generating and require confirmation in larger prospective multicenter cohorts.

## 5 Conclusion

Plasma KL-6 was associated with pulmonary disease severity before treatment and showed longitudinal changes during follow-up under enzyme replacement therapy in this small ASMD type B cohort. These preliminary findings support prospective multicenter evaluation of KL-6 as a non-invasive biomarker of alveolar epithelial injury, pulmonary disease activity, and treatment-associated change in ASMD.

## CRediT authorship contribution statement

**Karla Cifuentes-Uribe:** Writing – original draft, Data curation. **Nathalie Guffon:** Writing – review & editing, Supervision. **Lucie Boulière:** Formal analysis, Data curation. **Ségolène Turquier:** Validation, Formal analysis, Data curation. **Cécile Acquaviva-Bourdain:** Visualization. **Roseline Froissart:** Validation, Supervision. **Magali Pettazzoni:** Writing – review & editing, Supervision, Project administration, Methodology, Conceptualization.

## Declaration of competing interest

The authors declare that they have no known competing financial interests or personal relationships that could have appeared to influence the work reported in this paper.

## Data Availability

Data will be made available on request.
